# Conjunctival Reconstruction with Progenitor Cell-Derived Autologous Epidermal Sheets in Rhesus Monkey

**DOI:** 10.1371/journal.pone.0025713

**Published:** 2011-11-11

**Authors:** Rong Lu, Xinchun Zhang, Danping Huang, Bing Huang, Nan Gao, Zhichong Wang, Jian Ge

**Affiliations:** 1 State Key laboratory of Ophthalmology, Zhongshan Ophthalmic Center, Sun Yat-Sen University, Guangzhou, China; 2 Department of Prosthodontics, Hospital of Stomatology, Guanghua College of Stomatology, Sun Yat-sen University, Guangzhou, China; Universidade Federal do Rio de Janeiro, Brazil

## Abstract

Severe ocular surface diseases are some of the most challenging problems that the clinician faces today. Conventional management is generally unsatisfactory, and the long-term ocular consequences of these conditions are devastating. It is significantly important to find a substitute for conjunctival epithelial cells. This study was to explore the possibility of progenitor cell-derived epidermal sheets on denuded amniotic membrane to reconstruct ocular surface of conjunctiva damaged monkeys. We isolated epidermal progenitor cells of rhesus monkeys by type IV collagen adhesion, and then expanded progenitor cell-derived epidermal sheets on denuded amniotic membrane ex vivo. At 3 weeks after the conjunctiva injury, the damaged ocular surface of four monkeys was surgically reconstructed by transplanting the autologous cultivated epidermal progenitor cells. At 2 weeks after surgery, transplants were removed and examined with Hematoxylin-eosin staining, Periodic acid Schiff staining, immunofluorescent staining, scanning and transmission electron microscopy. Histological examination of transplanted sheets revealed that the cell sheets were healthy alive, adhered well to the denuded amniotic membrane, and had several layers of epithelial cells. Electron microscopy showed that the epithelial cells were very similar in appearance to those of normal conjunctival epithelium, even without goblet cell detected. Epithelial cells of transplants had numerous desmosomal junctions and were attached to the amniotic membrane with hemidesmosomes. Immunohistochemistry confirmed the presence of the conjunctival specific markers, mucin 4 and keratin 4, in the transplanted epidermal progenitor cells. In conclusion, our present study successfully reconstructed conjunctiva with autologous transplantation of progenitor cell-derived epidermal sheets on denuded AM in conjunctival damaged monkeys, which is the first step toward assessing the use of autologous transplantation of progenitor cells of nonocular surface origin. Epidermal progenitor cells could be provided as a new substitute for conjunctival epithelial cells to overcome the problems of autologous conjunctiva shortage.

## Introduction

The normal ocular surface is covered with highly specialized corneal and conjunctival epithelia, which are formed by two phenotypically different types of epithelial cells [1]. The cornea is covered by nonkeratinized, stratified epithelium that is responsible for maintaining ocular surface integrity and is essential for vision [2]. The conjunctival epithelium is well vascularized and consists of loosely organized cell layers. As an important component of the ocular surface, the conjunctiva plays a critical role in supporting the niche of the ocular surface and ensuring continued clarity and survival of the corneal epithelium and stroma [3], [4]. Severe ocular surface diseases and injuries – such as Stevens-Johnson syndrome, ocular cicatricial pemphigoid, severe microbial infection, and chemical or thermal burn – can damage conjunctival epithelium [5]–[7].

Many attempts have been made to establish a surgical treatment for severe ocular surface diseases. In such diseases, because iatrogenic injury to the remaining ocular surface and shortage of autograft implants in binocular disease patients, the alternative surgical treatment with amniotic membrane (AM) transplantation has been developed to improve the outcome of ocular surface reconstruction [8]–[11]. However, for severe ocular surface disease without normal conjunctival progenitor cell retained, normally AM transplantation couldn't obtain satisfied results. The most recently developed treatment for these diseases involves the transplantation of cultivated conjunctival epithelial cell for reconstructing the ocular surface after ocular surface damage [12], [13]. Nevertheless, in patients with severe conjunctival disease, there is often a shortage of normal conjunctival epithelium. Therefore, this method has limitations similar to those of autograft transplantation.

Our previous research found a small progenitor cell subpopulation isolated from epidermis of rhesus monkey showed dramatic cell plasticity, which changed to express conjunctival epithelial cell phenotype after co-cultured with conjunctival epithelial cells in vitro [14]. Similar research of our group also revealed that epidermal progenitor cells could be induced to differentiate into corneal epithelial cells in rabbits in vitro [15]. The results of other researchers also confirmed that epidermal progenitor cells could be differentiated into neurons, vertebrae, liver tissue, and brain tissue [16], [17]. All the researches suggest epidermal progenitor cells have the properties to differentiate the daughter cells of non skin origin.

This article describes an attempt to overcome the problems of allogeneic transplantation by using epidermal progenitor cells as a substitute for conjunctival epithelial cells. We isolated epidermal progenitor cells of rhesus monkey by type IV collagen adhesion, then expanded epidermal progenitor cells on AM ex vivo. We then transplanted the autologous cultivated progenitor cell-derived epidermal sheets onto the ocular surfaces of conjunctiva damaged monkeys and evaluated the survival of the tissue. This study is a first step toward assessing the use of autologous transplantation of epidermal progenitor cells of nonocular surface origin.

## Materials and Methods

### Animals

All animal use followed the guidelines of the Weatherall Report on the use of non-human primates in research and was approved before implementation by the Institutional Animal Care and Use Committee (IACUC) of Zhongshan Ophthalmic Center, Sun Yat-sen University (2010-030). The animals were housed under conditions approved by the Association for the Assessment and Accreditation of Laboratory Animal Care International. Four healthy Rhesus monkeys (*Macaca mulatta*, male, over 2-year old) were used for these researches (Files S1 and S2). Animals were maintained in biological security level 3 animal facilities and clinical examinations were performed regularly. Activities related to animal care were performed as per standard Sun Yat-sen National Primate Research Center operating procedures. All animals were negative for simian retrovirus (SRV) and simian T cell leukemia virus (STLV). An ocular-surface injury was created in one eye of each of the adult rhesus monkeys by excising all the bulbar and fornix conjunctival tissue. Antibiotic eye drops (0.5% Tobramycin) and intramuscular gentamicin (1 mg/kg) was administered after surgery.

### Materials and reagents

Cell culture medium was consisted of DMEM/F12 (V∶V = 3∶1) medium supplemented with 15% fetal bovine serum, 2 mM glutamine, 10 ng/ml hr-EGF, 10 ng/ml hr-bFGF, 10^−10^ mol/L cholera toxin, 5 µg/ml insulin, 100 U/ml penicillin, and 1000 ug/ml streptomycin, 0.4 µg/ml Hydrocortisone, 1.8×10^−4^ mol/L adenine, 5 µg/ml transferrin.

Cell culture dishes, plates, centrifuge tubes, and other plastic ware were purchased from Becton Dickinson and Company (Franklin Lakes, NJ). Nunc Lab-Tec II eight-chamber slides were from Nalge Nunc International Corp (Naperville, IL). Fetal bovine serum (FBS) was from Hyclone (Logan, UT). Dulbecco modified Eagle's medium (DMEM), Ham F-12, Keratinocyte-SFM (KSFM) and Defined KSFM (D-KSFM), amphotericin B, gentamicin, 0.25% trypsin/EDTA solution were from Invitrogen Corp (Carlsbad, CA). Mouse monoclonal antibody (mAb) against β1 Integrin was from BD Biosciences (San Jose, CA). Rabbit antibody anti MUC4 and keratin 15 were from Santa Cruz Biotechnology, Inc (Santa Cruz, CA). Mouse antibodies anti keratin 4 were from Boster Bio-engineering limited company, China. Mouse antibodies anti MUC5AC were from Millipore (Billerica, MA) Fluorescein Alexa-Fluor 488/594 conjugated secondary antibodies (Goat anti-mouse IgG or Goat anti-rabbit) were from Sigma-Aldrich (St. Louis, MO). Ready-To-Go You-Prime First-Strand Beads were from GE Healthcare (Piscataway, NJ). Type IV collagen, Human insulin, transferrin, sodium selenite, hydrocortisone, epidermal growth factor (EGF), cholera toxin A subunit, propidium iodide (PI), Hoechst and all other reagents came from Sigma-Aldrich (St. Louis, MO).

### Isolating and culturing rhesus monkey epidermal progenitor cells

Primary monkey epidermal cells were cultured from monkey skin biopsy specimens using a previously described protocol [14], [15]. In brief, after the monkey was anesthetized, about 5×5 cm^2^ of skin biopsy specimens were excised from the back of monkey and immediately immersed into phosphate-buffered saline (PBS) containing 1000 U/ml penicillin for 1 hour at room temperature (RT). And then the monkeys' back wounds were closed with 4–0 suture. The biopsy was finely removing the subepidermal tissue, cut into 2×2 mm^2^ each. Two pieces with the epithelium side up were directly put into a well of 6-well plate. The cell medium was used for cultures at 37°C under 5% CO2 and 95% humidity. The media were changed every 2–3 days.

Keratinocyte growth was carefully observed and photographed through a Nikon TE200 inverted phase microscope with a Nikon DXM1200 digital camera. Only the epithelial cultures without visible fibroblast contamination were used for this study. When grown to 90% confluence, the cultures were photographed and trypsinized with 0.25% trypsin/0.03% EDTA, and the cells were seeded into a new plate at a density of 2×10^4^ cells/cm^2^ for serial passages.

For isolation of progenitor cells that are enriched with putative epidermal progenitor cells, the cultured keratinocytes were allowed to attach to a collagen IV coated dish at 37°C for 20 minutes according to a procedure developed by Jones [18]. Briefly, the 3-passage cells were trypsinized and centrifuged for 5 minutes at 1000 rpm, and the resultant cell pellet was re-suspended in culture medium. Cells were transferred to a new flask that was coated with type IV collagen for 20 minutes at 3×10^4^ cells/cm^2^ under conditions of 95%O_2_–5%CO_2_ at 37°C. Then, the medium and any cells that did not adhere were discarded. The flask was washed gently with PBS once. The attached epidermal cells in 20 minutes were designated as rapid adherent cells (RAC). The isolated cell population was subjected to total RNA extraction for RT-PCR, immunofluorescent staining and flow cytometry to identify epidermal progenitor cells.

### Colony forming efficiency (CFE) and growth capacity

To evaluate proliferative capacity of rapid adherent cells of monkey epidermis, the CFE was assessed using a previous method [19] with modification. Rapid adherent cells isolated with type IV collagen were seeded in triplicate at 500 cells/cm^2^ into six-well culture plates with 3T3 fibroblasts as a feeder layer. Colonies with more than eight viable cells were counted manually under an inverted phase microscopy at days 8. Experiment was repeated at least three times. The CFE was calculated as a percentage of the number of colonies generated by the number of epithelial cells plated in a well. The growth capacity was evaluated on day 8 when cultured cells were stained with 1% rhodamine.

### Rapid adherent cells (RAC) cultured on denuded amniotic membrane

Human tissue was handled according to the Declaration of Helsinki. Preserved human AM was kindly provided by Guangdong Eye Bank (Guangzhou, China). AM was preserved according to the method described by Tseng [20]. Briefly, AMs derived from cesarean section placentas were rinsed in PBS containing 100 U/mL penicillin with 0.2 mg/mL streptomycin and stored in a solution of 50% DMEM and 50% glycerol at −80°C for at least 3 months. With this preservation method, both amniotic epithelium and stromal mesenchymal cells lose their viability and proliferative capacity. After thawing at room temperature, AM with the epithelial side facing up was fastened onto a culture insert, and treated with 0.1% sterile EDTA solution for 30 minutes and then gently scrubbed, to remove the amniotic epithelium without breaking the underlying basement membrane. With this method, 90% to 100% of the epithelium could be removed.

Isolated RACs were seeded on denuded amniotic membrane at 1×10^5^cells/cm^2^ and incubated at 37°C under 5% CO2 and 95% humidity, and the medium was changed every day. At 4 days after cultured on AM, the progenitor cell-derived sheets were used for autologous ocular surface reconstruction.

### Ocular surface injury and progenitor cell-derived autologous epidermal sheets transplantation

An ocular surface injury was created in one eye of each of the four adult rhesus monkeys by excising all the bulbal and fornix conjunctival tissue.

At 3 weeks after the ocular surface injury, the scared conjunctivae of the four rhesus monkeys were surgically reconstructed by transplanting autologous epidermal progenitor cells cultivated on AM. In all cases, the damaged ocular surface was carefully excised under anesthesia. All animals which epidermal progenitor cells had been placed in culture 4 days earlier received autologous cultivated epidermal progenitor cells on AM. All monkeys received the progenitor cell-derived epidermal sheets as a 6×12 mm^2^ of AM. The sheets were sutured to upper sclera surface with 10-0 nylon sutures with cells facing up. After surgery, topical antibiotics (0.5% Tobramycin) and steroids (0.1% Dexamethasone) were applied three times daily. For our experimental controls, four eyes received AM transplants onto upper sclera, which were preserved at −80°C over 3 months.

At 2 weeks after surgery, all transplants, including progenitor cell-derived epidermal sheets and preserved AM transplants, were removed and embedded in optimal cutting temperature (OCT) compound for cryosectioning.

### Hematoxylin-eosin staining and Periodic acid Schiff staining

Tissues were fixed in 10% formalin and embedded in paraffin. Serial sections (five µm thick) of cultivated epithelia were generated. Sections were deparaffinized, rehydrated with distilled water, and stained with hematoxylin and eosin. Sections were observed under a light microscope.

Paraffin sections were deparaffinized, rehydrated, and then treated with periodic acid for 5 min. The sections were washed, treated with Schiff's reagent for 15 min, and stained with hematoxylin.

### Immunofluorescent staining

Immunofluorescent staining was performed following a previously reported method [21]. In brief, transplant frozen sections, cultured epidermal progenitor cells were fixed with cold methanol at 4°C for 10 minutes. Cultured cells were permeated with 0.2%Triton X-100 in PBS at room temperature for 10 minutes. After blocking with 10% normal goat serum in PBS for 30 minutes, primary mAbs against keratin 15 (1∶100), *β*1 Integrin (1∶1000), keratin 4 (1∶100), mucin 4 (1∶100) and MUC5AC (1∶1000) were applied and incubated for 2 hours at room temperature. A secondary antibody, Alexa Fluor 488-conjugated Goat anti-mouse IgG (1∶300) or 594-conjugated goat anti-rabbit IgG (1∶300), were then applied and incubated in a dark chamber for 1 hour, or followed by counterstaining PI (2 µg/ml in PBS) or Hoechst DNA-binding dye for 5 minutes. After washing with PBS, Antifade Gel/Mount and a cover slip were applied. Sections were examined and photographed with the LSCM (LSM 510, Zeiss, Thornwood, NY).

### Total RNA extraction and reverse transcriptase–polymerase chain reaction (RT-PCR)

RT-PCR was used to examine expression of *β*1 Integrin and cytokeratin 15 in RACs, as an easy kit according to manufacturer's protocol (Qiagen, Germany). The RNA was quantified by its absorption at 260 nm and stored at −80°C before use. With a housekeeping gene, glyceraldehyde-3-phosphate dehydrogenase (GAPDH), as internal control, the mRNA expression of different molecular markers by isolated progenitor cells were analyzed by semi quantitative reverse transcriptase-polymerase chain reaction (RT-PCR) as described in our previous reports [21]. Briefly, first-strand cDNAs were synthesized from 0.5 µg of total RNA with murine leukemia virus reverse transcriptase. PCR amplification of the first-strand cDNAs was performed with specific primer pairs, designed from published human gene sequences for different markers in a GeneAmp PCR System 9700 (Applied Biosystems). Semi-quantitative RT-PCR was established by terminating reactions at intervals of 20, 24, 28, 32, 36, and 40 cycles for each primer pair to ensure that the PCR products formed were within the linear portion of the amplification curve. The fidelity of the RT-PCR products was verified by comparing their size to the expected cDNA bands and by sequencing the PCR products.

### Scanning electron microscopy

At 2 weeks after transplantation, transplants were removed from ocular surface and washed two times with cacodylate buffer (pH 7.3). The cells were fixed with cacodylate buffered Karnovsky's solution, post fixed in 1% osmium tetroxide and embedded in Epon, according to standard scanning electron microscopy techniques. Cells were mounted on aluminium stubs, sputter-coated with gold and examined with a scanning electron microscope (JEOL-JSM-T300 Scanning microscope).

### Transmission electron microscopy

The specimens, about 1 mm cubes, were excised from transplants, and fixed for 1 hour in 3% glutaraldehyde buffered to pH 7.2 with 0.01 M PIPES (piperazine-N,N′-bis[2-ethane sulfonic acid]). They were rinsed in buffer and post-fixed in PIPES-buffered osmium tetroxide (pH 7.2) for 1 hour at room temperature, then rinsed in several changes of distilled water and dehydrated through a graded series of ethanol. The dehydrated tissues were incubated in two 45-minute changes of propylene oxide followed by a 1∶1 mixture of propylene oxide and Spurr's resin for one and a half hours. The tissue pieces were then incubated in pure resin for one and a half hours, after which they were transferred to fresh resin in block molds and allowed to cure at 60°C overnight. One µm thick sections cut from the hardened blocks were mounted on glass slides, stained with an alcoholic solution of toluidine blue and basic fuchsin, and examined under the light microscope. Areas of interest were trimmed and 60 nm sections were cut and mounted on copper grids (300 mesh). The grids were stained with uranyl acetate and lead citrate and photographed with a Zeiss EM- 900 transmission electron microscope (Zeiss; Peabody, MA; http://www.zeiss.com). Photographs were taken with Kodak 4489 Electron Microscope film (Eastman Kodak; Rochester, NY; http://www.kodak.com).

### Flow Cytometry

Rapid adherent cells with type IV collagen adhesion were detached from dishes for flow cytometry analysis by first washing twice in DPBS for 5 min at 37°C. Cells were then incubated in 0.05% trypsin-EDTA for 10 min at 37°C, followed by neutralization using FACS buffer (DBPS, 2% FBS). Cells were filtered through a 40-µm cell strainer and centrifuged 5 min at 300 g, supernatant discarded, and the pellet was resuspended in DPBS with 1% paraformaldehyde (Sigma) for fixation for 30 min at room temperature. Cells, at 5×10^6^ cells/ml, were washed twice in FACS buffer plus 0.1% Triton X-100 and centrifuged. Primary antibody, mouse monoclonal to β1 Integrin was diluted 1∶500, and rabbit antibody against keratin 15 was diluted 1∶50 in 300 µL per sample FACS buffer. Samples were incubated with primary antibody overnight at 4°C. Cells were washed twice in FACS buffer. Samples were incubated 30 min with secondary antibody in the dark at room temperature, then washed twice in FACS buffer. Samples were centrifuged, re-suspended in 500 µL per sample FACS buffer, transferred to flow cytometry tubes, and stored on ice until analysis. Data were collected on a FACS Caliber flow cytometer (Beckton Dickinson) and analysis performed using CellQuest (Beckton Dickinson) software. All cells were gated according to light scatter and fluorescence.

## Results

### Isolation and identification of monkey epidermal progenitor cells

**Figure 1:**
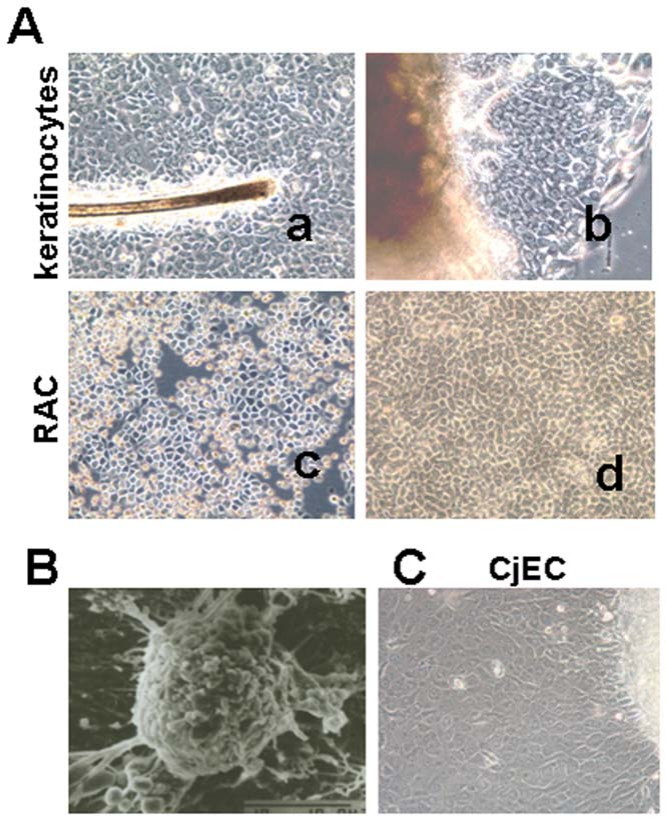
Growth pattern of monkey keratinocytes and Rapid Adherent Cells (RAC). A. Representative phase images of primary monkey keratinocytes (P0) and RAC. a. keratinocytes (P0) growing around hair follicle and almost reaching confluence (×200); b. keratinocytes growing from explants (×200); c. RAC adhered on Type IV collagen coated culture flask within 20 minutes (×200); d. RAC reaching confluence (×200); B. Scanning electron microscopy photography of RAC (×3500); C. Representative phase image of conjunctival epithelial cells growing from explant (×200).

The primary monkey keratinocytes cultures showed a mosaic of stratified epithelial cells, with heterogeneous morphologies and different sizes, including very small cells and large cells, as Fig. 1A-a showed. The keratinocytes spreading out from explants expanded like a sheet and stratified after reaching confluence (Fig. 1A–Fig. 1b). The 3-passage keratinocytes were isolated with type IV collagen for 20 minutes. Fig. 1A, 1b, 1c and 1A, 1b, 1c, 1d showed that the isolated rapid adherent cells (RAC) which appeared typical epithelial cell morphology with a small round or oval appearance. With scanning electron microscopy, RAC showed typical stem cell morphology of smaller cell size with big nucleus/cytoplasm ratio and abundant of microvilli on the surface of the cell (Fig. 1B). In comparison to monkey conjunctival epithelial cells (Fig. 1C), skin keratinocytes showed more pigmented cytoplasm and tighter cell junctions.

**Figure 2:**
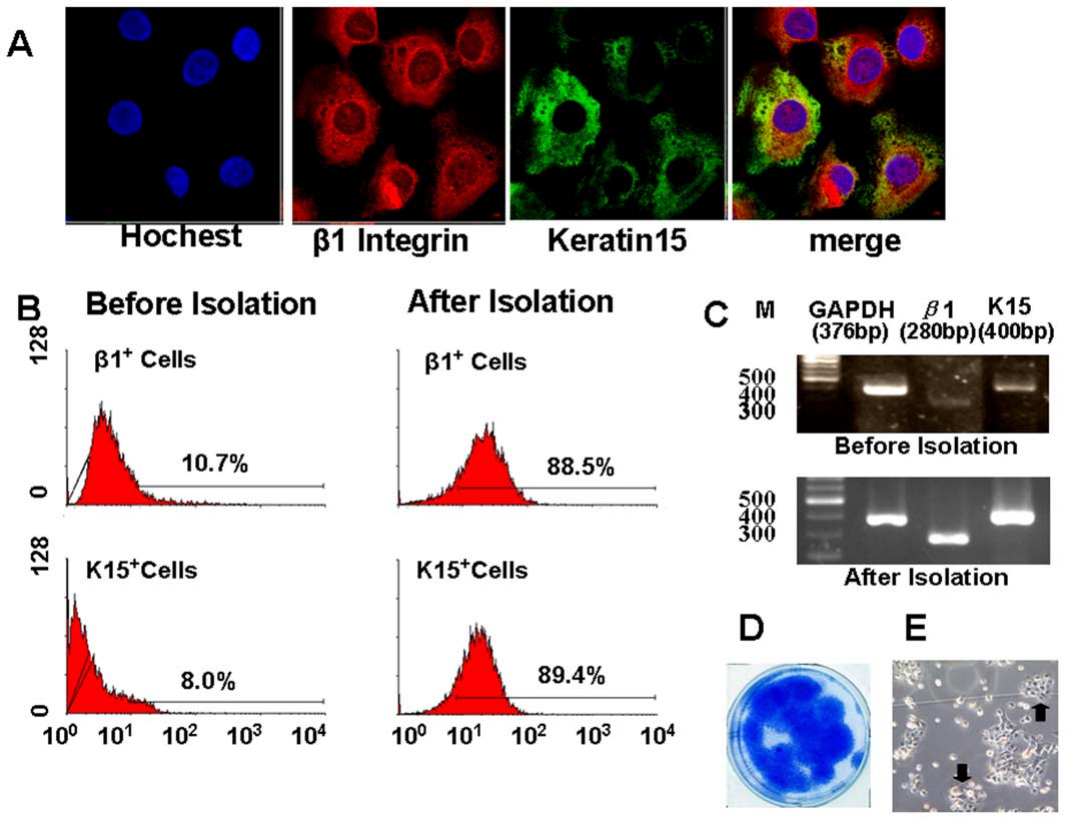
Growth capacity and cell phenotype of RAC isolated with Type IV collagen adhesion. A. Representative images of immunofluorescent staining of epidermal stem cell associated markers, β1 Integrin (red) and Keratin 15 (green) with Hoechst (blue) counterstaining; B. Flow cytometry analysis of these markers by keratinocytes before and after isolation; C. RT-PCR image showing the expression levels (mRNA) of these markers by keratinocytes before and after isolation; D. Representative cultures stained with 1% rhodamine on day 8 showing growth capacity of RAC; E. Representative phase images of RAC on day 3 showing single colonies.

To evaluate their clonal growth capacity, RAC of monkey epidermis were seeded at a density of 500 cells/cm^2^ with a 3T3 fibroblast feeder layer to assess colony forming efficiency (CFE). RAC of monkey epidermis generated a significant number of colonies. As shown in Fig. 2D, the CFE of RAC reached 33.2±1.8% on day 8.

To evaluate the phenotype of the isolated epidermal progenitor cells, RAC isolated by type IV collagen was seeded into wells of eight-chamber culture slides for immunofluorescent staining with antibodies against epidermal stem cell associated markers, *β*1 Integrin and keratin 15. RAC were strongly positive for *β*1 Integrin and keratin 15 (Fig. 2A). To verify the identification of epidermal progenitor cells we used isolated RAC to perform double immunostaining between *β*1 Integrin and keratin 15, the proposed epidermal stem cell associated markers. The results showed both *β*1 Integrin and keratin 15 were strongly expressed by most of RAC (Fig. 2A).

These results were further substantiated by flow cytometry. Flow cytometry results disclosed different expression ratio of stem cell associated markers, keratin 15 and *β*1 Integrin, in type IV collagen isolated RAC and all of the keratinocytes. Fig. 2B showed *β*1 Integrin positive cells in all of the cells, which was 10.7% before isolation and 88.5% after isolation. The percentage of keratin 15 positive cells before isolation was 8% and 89.4% after isolation (Fig. 2B).

The expression of these markers was further confirmed at the transcriptional level by reverse transcription and semi quantitative RT-PCR, as shown in Fig. 2C. With GAPDH as an internal control, the mRNA expression of epidermal stem cell associated markers, *β*1 Integrin and keratin 15, were detected significantly in epidermal progenitor cells isolated with type IV collagen.

### Cultivation of progenitor cell-derived epidermal sheets on denuded AM and autologous transplanted to ocular surface

**Figure 3:**
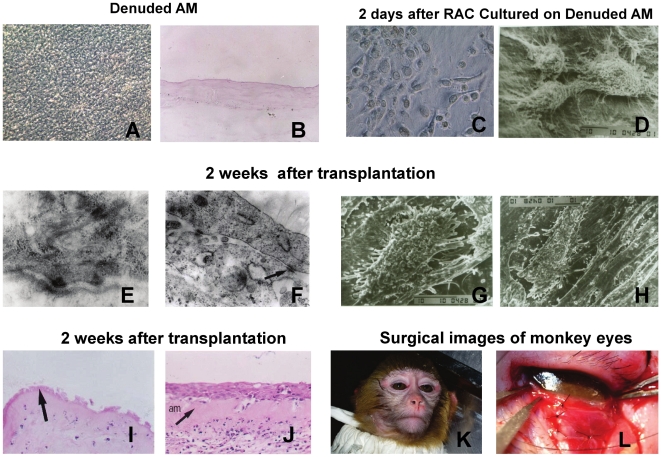
Cultivation of epidermal progenitor cell-derived sheets on denuded AM and autologous transplanted to ocular surface. A and B. Images of Denuded AM. A. Representative phase image (×100); B. Hematoxylin and eosin staining image (×100); C and D. Representative images of epidermal progenitor cells cultured on denuded AM at 2 days. C. Representative phase image showing epidermal progenitor cells form colonies on AM within 48 hours (×400); D. Scanning electron microscopy photography showing epidermal progenitor cells adhered tightly with AM (×2000); E to H. Electron microscopy photography of progenitor cell-derived epidermal sheets at 2 week after transplantation. E. TEM photography showed hemidesmosomes between basal cells and AM (×15000); F. Adjacent cells were joined with numerous desmosomal junctions (×13000); G and H. SEM photography showed the cells appeared to be in good condition and were closely attached to each other with tightly fitting cell junctions (×3500); I. HE staining image of transplanted preserved AM in controls, which showed discontinuous monolayler epithelium, and no nuclei found in the epithelial cells (×200); J. HE staining image of autologous transplantation of progenitor cell-derived epidermal sheets, which showed several layers of healthy epithelial cells on AM (×200); K and L. Surgical images of monkey eyes.

As figure 3A and B shown, with 0.1% EDTA solution treatment and gently scrubbed, over 95% of the amniotic epithelium was removed without breaking the underlying basement membrane. Epidermal progenitor cells of rhesus monkey began to form colonies on the denuded AM within 48 hours, as figure 3C and D shown.

At 3 weeks after the ocular surface injury, ocular surface scars covered the damaged conjunctival surface in all 4 monkeys with considerable neovascularization and subconjunctival inflammation evident. The extent of injury was similar in all animals.

After the removal of conjunctival scar tissue, we reconstructed the ocular surface with a cultivated autologous progenitor cell-derived epidermal sheet on AM or preserved AM. Within 2 weeks after transplantation, no signs of infection, bleeding, or sheet detachment were observed.

At 2 weeks after transplantation, ultrastructural examination revealed an architecture of well-structured, compact, multilayered cell sheets with the expected microstructures of the native cells, including microvilli, tight junctions, desmosomes, and hemidesmosomes. Such morphologic characteristics are similar to those of conjunctival epithelium in vivo. Transmission electron microscopy (TEM) was used to check the presence of cell-cell junctions and cell-basement membrane junctions. As shown in Figure 3F, cultured epidermal progenitor cells were able to form gap junctions and desmosomes with each other. Moreover, hemidesmosomes, the junctions between basal epithelial cells and the amniotic membrane, were clearly visible in culture sheets (Fig. 3E). SEM examination of the cultivated epidermal progenitor cells revealed a continuous layer of flat, polygonal epithelial cells (Fig. 3G). These cells appeared healthy and well formed with distinct cell boundaries.

Histological examination of transplanted sheets at 2 weeks after surgery revealed that the sheets adhered well to the denuded AM. Superficial cells of the transplanted sheets had nuclei, indicating that they were indeed nonkeratinized mucosal epithelial cells (Fig. 3K). With hematoxylin-eosin (HE) staining, the autologous transplanted progenitor cell-derived epidermal sheets showed multilayered stratification, well differentiated epithelium (Fig. 3K), and appeared very similar to normal conjunctival epithelium. No goblet cells were seen in these cultures by periodic acid Schiff (PAS) staining (data not shown). The control animals that had undergone transplantation of preserved AM showed discontinuous monolayer epithelium (Fig. 3I). No nuclei were found in the epithelial cells with HE staining, indicating that the epithelium of transplanted preserved AM were not alive.

**Figure 4:**
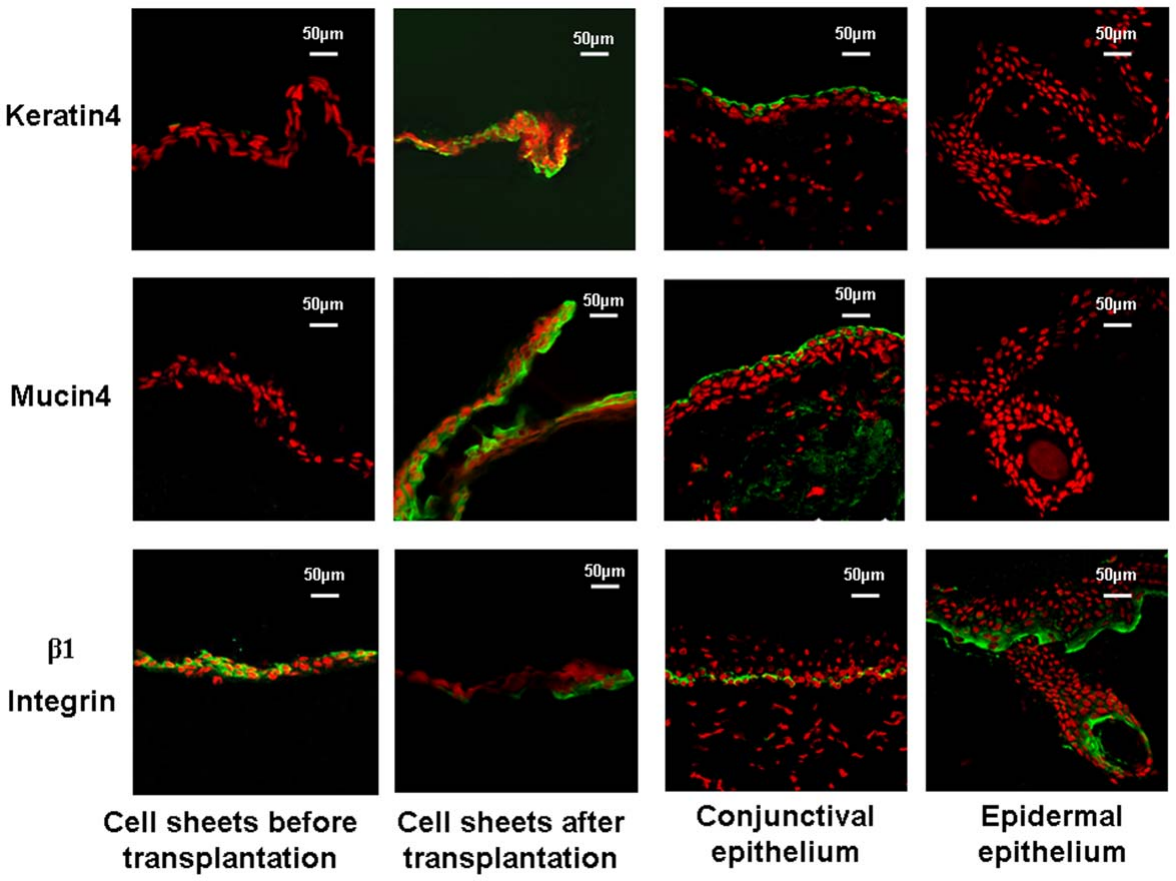
Representative images showing immunofluorescent staining of MUC4, keratin4 or β1 Integrin (green) with Propidium Iodide (PI) (red) counterstaining of cell sheets before transplantion and 2 weeks after transplantation, normal conjunctival epithelium and epidermal epithelium.

Progenitor cell-derived epidermal sheets on AM were found losting epidermal progenitor cell property gradually. With immunofluorescent staining, at 2 weeks after transplantation autologous transplanted progenitor cell-derived epidermal sheets showed a few *β*1 Integrin positive cells (Fig. 4). While other conjunctival specific markers, keratin 4 and mucin 4 showed strongly positive in transplanted cell sheets. Even no MUC5AC positive goblet cell was detected (data not shown), the results suggested that after transplanted to ocular surface, epidermal progenitor cells had lost part of epidermal progenitor properties and changed to be conjunctiva-like epithelial cells.

## Discussion

Severe ocular surface diseases such as SJS and OCP are some of the most challenging problems that the clinician faces today [22], [23]. Conventional management is generally unsatisfactory, and the long-term ocular consequences of these conditions are devastating [24], [25]. In the past 10 years, surgical reconstruction of the ocular surface has been greatly advanced by the introduction of AM transplantation [26], [27]. However, for severe ocular surface disease without normal conjunctival progenitor cell retained, normally AM transplantation couldn't obtain satisfied results. In our present study, we explored the possibility of progenitor cell-derived epidermal sheets on AM to reconstruct ocular surface of conjunctiva damaged monkeys, which is the first step toward assessing the use of autologous transplantation of progenitor cells of nonocular surface origin.

The skin is the largest organ in the body. It consists of an outer layer, the epidermis and an inner layer, the dermis of mesodermal origin. The epidermis is a dynamic epithelium that is constantly renewed throughout life. A subpopulation of cell, epidermal progenitor cells are responsibility for the epidermis self-renew, which is located in the upper hair follicle (bulge) and patches of basal keratinocytes [28]–[30]. There are now a number of reports indicating that epidermal progenitor cells have differentiation potential outside the organ in which they reside [31], [32]. These characteristics suggest that epidermal progenitor cells could be an ideal substitute for conjunctival epithelial cells for use in ocular surface reconstruction.

Epidermal progenitor cells could be isolated by several methods, including methods based on cell surface markers, such as flow cytometry and immunomagnetic bead selection [33], [34]. We isolated monkey epidermal progenitor cells with type IV collagen adhesion, in the most effective procedure used to isolate epidermal progenitor cells [18], [30], [35], [36]. Type IV collagen, the *β*1 Integrin ligand, is responsible for the attachment of the basal layer of the epidermis to its underlying substratum, the basement membrane. Epidermal progenitor cells highly express *β*1 Integrin, which correlates with rapid adhesion to type IV collagen [29], [30]. Thus, keratinocytes can be sorted on the basis of whether they are rapidly (within 20 min.) or slowly adherent to type IV collagen. Additionally, using flow cytometry we analyzed *β*1 Integrin and keratin 15 expression in RAC and unsorted cells, and found the ratio of *β*1 Integrin and keratin 15 positive cells in RACs almost reaching 90%. With this study, we also proved that type IV collagen adhesion was effective for epidermal progenitor cells isolation.

We identified epidermal progenitor cell with its proliferative capacity and progenitor phenotype. β1 Integrin has been accepted as a basal cell marker that is associated with certain stem cell properties [30], [32], [33], [37]–[39]. High *β*1 Integrin expression is required to maintain keratinocytes in an undifferentiated state [40], [41]. *β*1 Integrin regulates the differentiation of stem cells through MAP kinase signaling [38], [39]. Integrin keeps cells in the right place in a tissue, and loss or alteration of integrin expression will make the stem cells undergo differentiation or apoptosis [17], [40], [42]. Often, a combination of several markers serves to identify epidermal progenitor cells, and *β*1 Integrin usually serves as the basis of the other characteristic markers [35]. Keratin 15 is another marker that recently has been found to be highly expressed in epidermal stem cells in the bulge region [34], [43]. In our present study, with molecular markers, *β*1 Integrin and keratin 15, we identified epidermal progenitor cells from morphology, RNA, and protein levels. Moreover, we analyzed the colony forming efficiency (CFE) of epidermal progenitor cells. CFE assay is usually used to monitor the proliferative capacity of progenitor cells. As figure 3 showed, the CFE of RAC reached 33.2±1.8%, consistent with the reported CFE in epidermal stem cells [44].

After the successful culture of epidermal progenitor cells on AM, we tried to reconstruct the damaged conjunctiva by transplantation of autologous cultivated progenitor cell-derived epidermal sheets, to test the viability of using these cells as a substitute for conjunctiva.

The results are of particular interest. SEM examination revealed that progenitor cell-derived epidermal sheets, after 2 weeks transplanted on ocular surface, appeared healthy and well formed with tightly opposed cell junctions. Some basal cells formed hemidesmosome between basal cell and denuded AM. And desmosomes were found to connect adjacent cell tightly. TEM examination confirmed that the progenitor cell-derived epidermal sheets were very similar in appearance to that of the conjunctival epithelium. Similar to the conjunctival epithelium, it had multilayered stratified cells. Both of SEM and TEM results indicate that our epidermal progenitor cells, after transplanted on ocular surface to replace conjunctiva, resembled normal conjunctival epithelial cells more closely.

Mucin 4 and keratin 4, are the most common markers for conjunctival non-goblet epithelial cells [45], [46]. Mucins are a group of high molecular weight glycoproteins consisting of a mucin core protein and O-linked carbohydrates [47], [48]. MUC 4 is a membrane-bound mucin. The mucin 4 gene is the predominant mucin gene expressed in the normal urothelium and it is also expressed in several normal tissues such as those in the trachea, lung, testis [49], [50], conjunctival and corneal epithelium [51], but has not been found in skin. Cytokeratins play an important structural and protective role in maintaining the integrity of the epithelium of the anterior segment of the eye. Keratin 4 reportedly, is a reliable marker for conjunctival epithelial cells [52]. Interestingly, after transplanted to replace conjunctiva, progenitor cell-derived epidermal sheets showed mucin 4 and keratin 4 positive with immunofluorescent staining (Fig. 4). The result confirms that after transplanted on ocular surface the epidermal progenitor cells have differentiated into conjunctiva-like epithelial cells other than any other cell type under the ocular surface niche.

In conclusion, our present study successfully reconstructed conjunctiva with autologous transplantation of progenitor cell-derived epidermal sheets on denuded AM in conjunctival damaged monkeys, which is the first step toward assessing the use of autologous transplantation of progenitor cells of nonocular surface origin. Epidermal progenitor cells could be provided as a new substitute for conjunctival epithelial cells to overcome the problems of autologous conjunctiva shortage.

## Supporting Information

File S1 (JPG)

File S2 **Ethics statements for rhesus monkeys used in this present research (in Chinese).**
(JPG)
