# Supplementary figures and images for: Conjunctival Reconstruction with Progenitor Cell-Derived Autologous Epidermal Sheets in Rhesus Monkey
Source: PLoS One. 2011 Nov 11;6(11):e25713. doi: 10.1371/journal.pone.0025713 (PMC3214019; doi:10.1371/journal.pone.0025713)

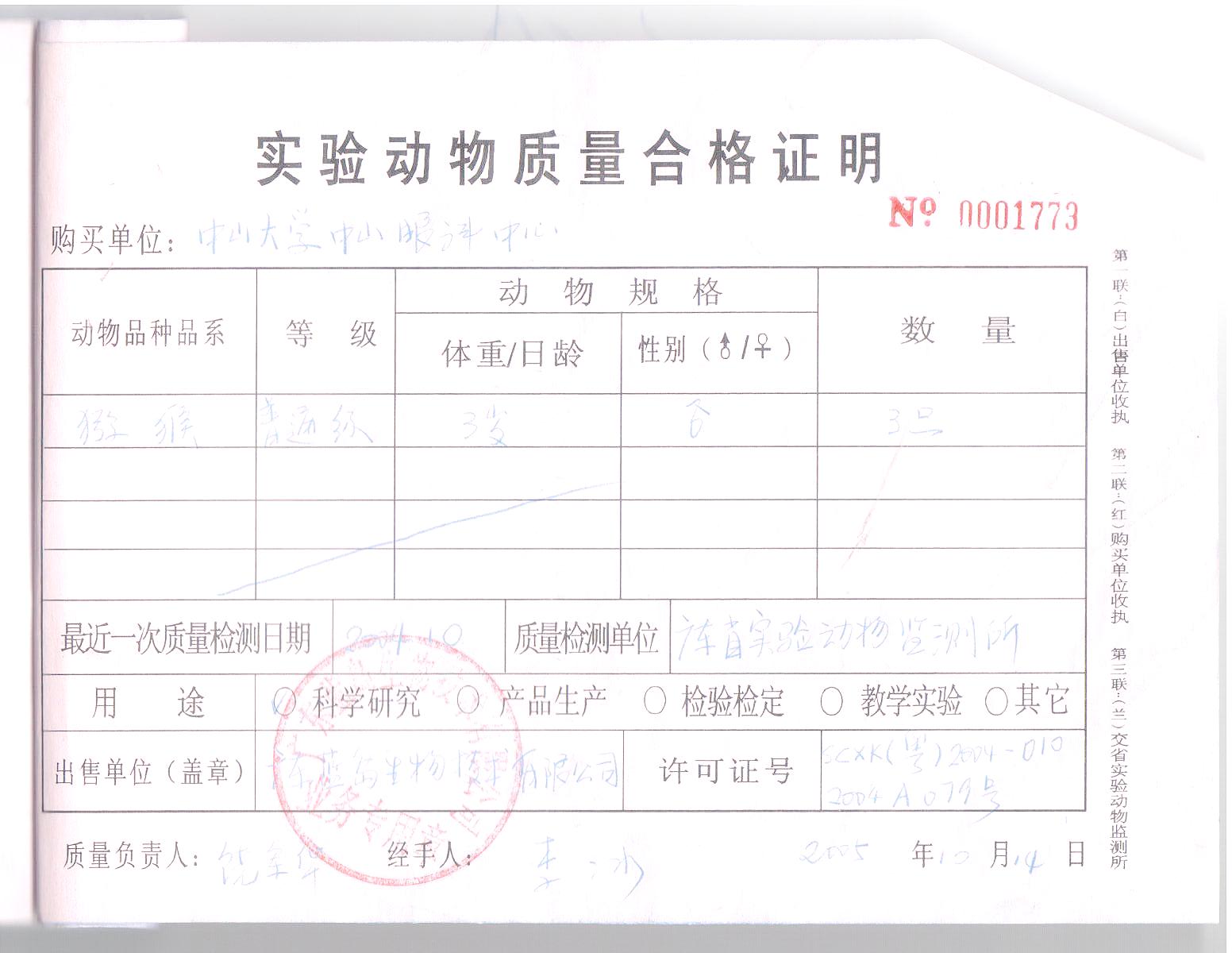

Supplement: File S1 — (JPG) [file pone.0025713.s001.jpg]

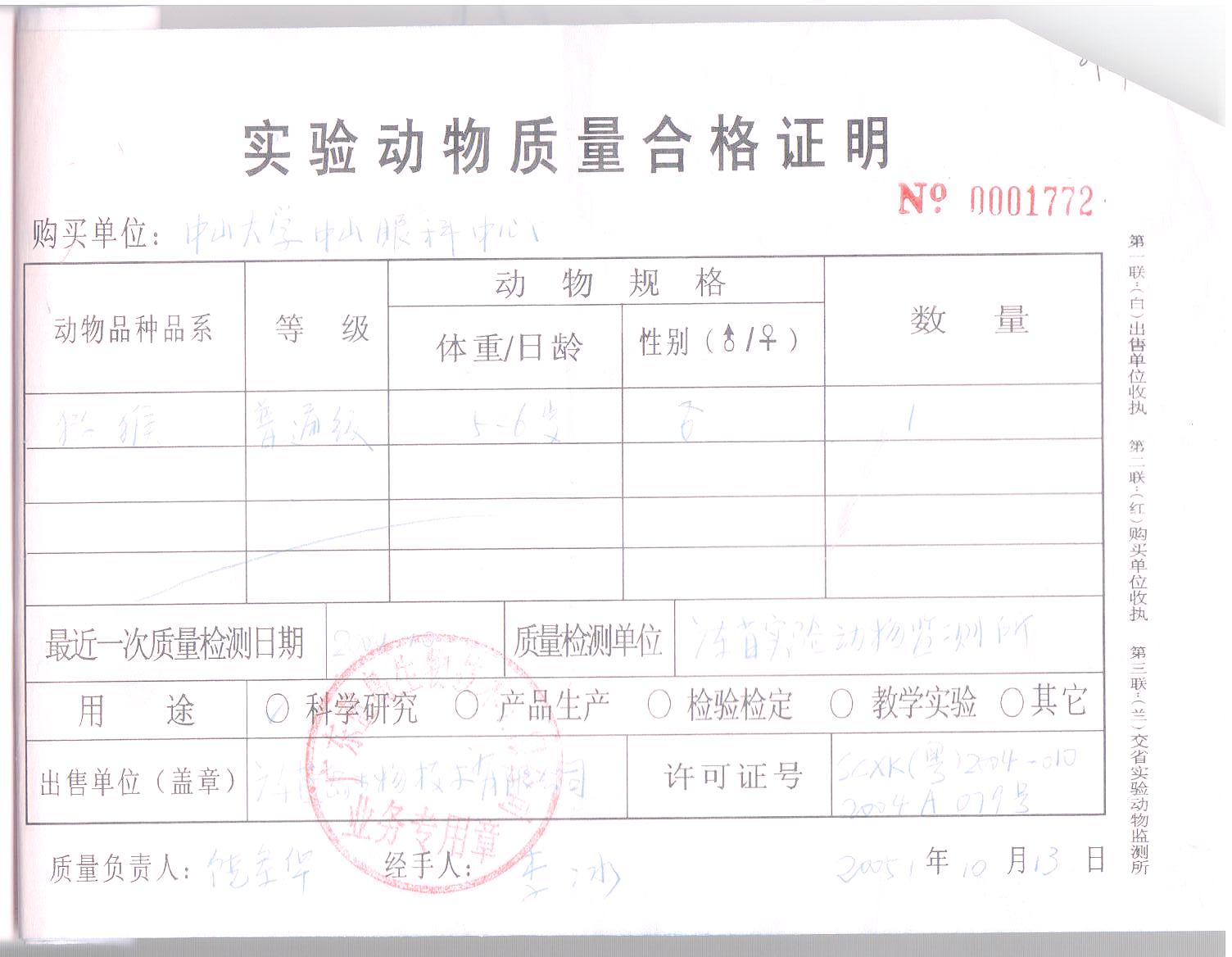

Supplement: File S2 — Ethics statements for rhesus monkeys used in this present research (in Chinese). (JPG) [file pone.0025713.s002.jpg]
